# Natural Transmission of Zoonotic *Babesia* spp. by *Ixodes ricinus* Ticks

**DOI:** 10.3201/eid1502.081247

**Published:** 2009-02

**Authors:** Claire A.M. Becker, Agnès Bouju-Albert, Maggy Jouglin, Alain Chauvin, Laurence Malandrin

**Affiliations:** Institut National de la Recherche Agronomique, Nantes, France (C.A.M. Becker, A. Bouju-Albert, M. Jouglin, A. Chauvin, L. Malandrin); Ecole Nationale Vétérinaire de Nantes, Nantes (C.A.M. Becker, A. Bouju-Albert, M. Jouglin, A. Chauvin, L. Malandrin)

**Keywords:** Babesia divergens, Babesia sp. EU1, zoonotic disease, tick, Ixodes ricinus, salivary glands, transmission, sporozoite, cell culture, dispatch

## Abstract

To determine characteristics of natural transmission of *Babesia* sp. EU1 and *B.* *divergens* by adult *Ixodes ricinus* ticks, we examined tick salivary gland contents. We found that *I. ricinus* is a competent vector for EU1 and that their sporozoites directly invade erythrocytes. We conclude that EU1 is naturally transmitted by *I.* *ricinus*.

*Ixodes ricinus* is a ubiquitous triphasic tick found commonly in Europe. This arthropod feeds on a wide variety of vertebrate hosts, including small rodents and wild and domestic ungulates. It is therefore a potential vector of numerous pathogens, such as bacteria, viruses, and parasites, mainly apicomplexans. Among these pathogens, 2 zoonotic *Babesia* species have been described in Europe: the well-known cattle parasite *Babesia* *divergens* (*1*) and the more recently reported roe deer parasite *Babesia* sp. EU1 (*2*–*4*). Biological transmission of *B.* *divergens* by *I. ricinus* ticks has been proven by in vivo experimental infections (*5*); however, quantitative transmission studies that visualize and quantify sporozoites have never been conducted. For *Babesia* sp. EU1, biological evidence of natural transmission by *I. ricinus* ticks is still lacking; its presence has been assessed only by DNA amplification from whole ticks (*4*,*6*–*8*). Therefore, to analyze transmission of zoonotic *Babesia* spp. by *I. ricinus* ticks, we visualized, isolated, and identified infectious sporozoites from dissected tick salivary glands, the transmitting organs.

## The Study

In 2008, ticks were collected from animals from 2 different biotopes where each *Babesia* species had been known to circulate: a farm on which a herd was infected with *B.* *divergens* and a reserve on which wild fauna were infected with *Babesia* sp. EU1. A dairy farm in La Verrie (Vendée, France) was selected as a favorable biotope for *B.* *divergens* transmission on the basis not only of the presence of numerous ticks on cows and in pastures in 2007 but also of the parasite circulation in the herd, attested by serologic testing (prevalence of 37.5% by immunofluorescence antibody test [IFAT]) and confirmed by its isolation from cattle erythrocytes (prevalence 25% by culture) (*9*). Of the cows tested by IFAT, 56% had positive results, which indicated that new infections from ticks were occurring within the herd. Because we assumed that sporozoite differentiation is stimulated by blood ingestion and because of experimental proof that female ticks can transmit *B.* *divergens* (*10*), we collected only adult ticks feeding on cows. The 324 collected ticks were morphologically identified as *I. ricinus* and weighed to estimate their repletion status (range 3–398 mg). Of these, 223 ticks (4.7–339 mg) were dissected under a stereomicroscope to isolate both salivary glands, which were subsequently crushed in 30 μL phosphate-buffered saline in a 1.5-mL microtube with an adapted pestle. A droplet of this suspension was deposited on an 18-well slide, stained with May-Grünwald-Giemsa, and examined under a light microscope. When parasites were seen, and for 41 additional negative samples within the same weight range, 5 μL of the infected suspension was added to the culture medium with bovine (*B.* *divergens* selective growth) or sheep (both species growth) erythrocytes, RPMI (Roswell Park Memorial Institute medium; Lonza, Basel, Switzerland), and 20% fetal calf serum (Lonza) in 96-well plates (*11*).

To identify the parasites, we directly sequenced the amplified 18S rDNA *Babesia* gene. PCR with Phusion High-Fidelity DNA Polymerase (Finnzymes, Espoo, Finland) was performed on extracted DNA (Wizard Genomic DNA Purification Kit; Promega, Madison, WI, USA) from the remaining crushed salivary gland suspensions (Bab primers GF2 and GR2, 540 bases long, variable part of the gene) (*4*) and from resulting parasitized erythrocytes (primers CryptoF and CryptoR, 1,727 bases long, complete gene) (*12*).

To confirm the identity of the infected ticks, we directly sequenced a variable part of the 16S rDNA mitochondrial gene of *Ixodes* ticks (310 bases long) (primers IrUp1 5′-TTGCTGTGGTATTTTGACTATAC-3′ and IrDo2 5′-AATTATTACGCTGTTATCCCTGA-3′). We used DNA extracted from salivary glands.

Microscopic observation of crushed salivary gland suspensions identified small pear-shaped elements in only 3 ticks; weights were 11.7, 25.3, and 277 mg. These millions of pyriform parasites were considered to be sporozoites (*13*): they measured about 2 μm in length and 1 μm in diameter (Figure, panel A). Only a few parasites had unusual forms, which suggests binary fission (Figure, panel B).

**Figure:**
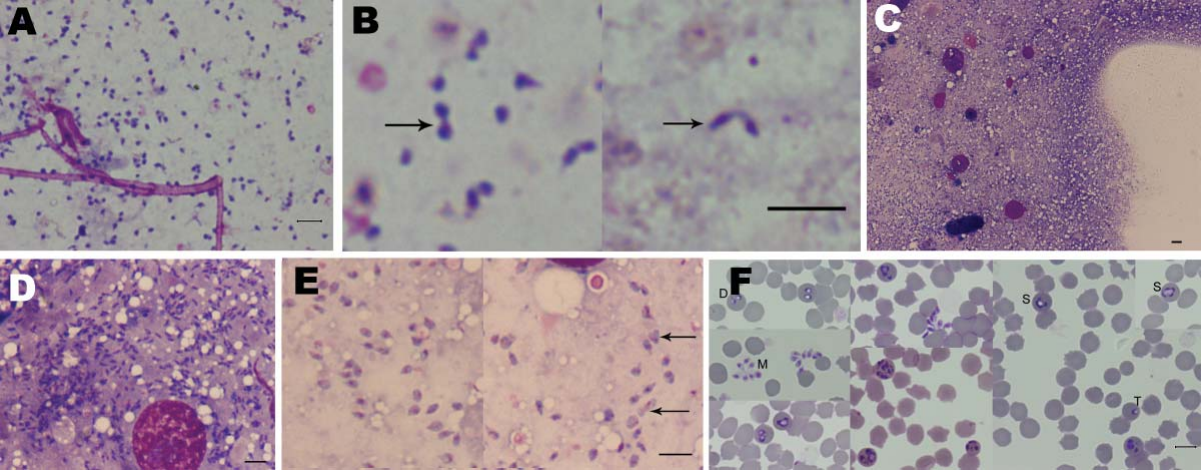
Microscopic appearance of *Babesia* sp. EU1 sporozoites isolated from tick salivary glands and of subsequent asexual development in erythrocytes. Sporozoites were stained with Giemsa and observed in the suspension of crushed salivary glands (A, B) and from salivary glands directly crushed between slides (C, D, E). Arrows indicate sporozoite dividing forms. A composite panel of asexual stages cultivated in sheep erythrocytes from these sporozoites is presented (F); developmental stages are indicated by letters (D, dividing stages; M, free merozoites; S, schizont-like form; T, trophozoite). Scale bars = 5 μm.

Development of intraerythrocytic parasites was observed, which proved the parasites’ capacity to directly infect erythrocytes. Of the 3 tick salivary glands containing pear-shaped elements, 3 days after inoculation onto a culture, ≈1/10,000 erythrocytes was infected. Only sheep erythrocytes were invaded, which suggests infection with *Babesia* sp. EU1. From these 150-μL starting wells, 10-mL amplified cultures (10% parasitized erythrocytes) could be established within 1 month (Figure, panel F). Typical *Babesiidae* developmental forms (trophozoite, dividing stages, and free merozoites) were observed, as were more atypical schizont-like parasites, which seemed to produce numerous merozoites. When sporozoites were not observed, parasites were never observed in the cultures of either bovine or sheep erythrocytes.

PCR amplification, sequencing, and comparison with *Babesia* spp. 18S rDNA gene (BLAST [www.ncbi.nlm.nih.gov/blast/Blast.cgi] search in GenBank) showed the sequences to be 100% identical to the *Babesia* sp. EU1 sequence (AY046575) for the 3 infected ticks (sporozoites and culture). The partial (sporozoites) and complete (culture) 18S rDNA sequences obtained have been deposited in GenBank, accession nos. FJ215872 and FJ215873. Identity of the ticks was confirmed by sequence analysis and comparison with the 16S rDNA *I. ricinus* gene (U14154).

For the wild fauna reserve, we used the same approach. At the reserve of Chizé (Deux-Sèvres, France), where high prevalence *Babesia* sp. EU1 has been described (*4*), we captured 18 roe deer, then collected and analyzed blood samples from them. Presence of *Babesia* sp. EU1 was attested by culture of samples from 4 of the deer. For 31 female ticks, half of the ticks were processed as previously described, and the salivary glands of the other half were simply crushed between 2 slides so parasites could be better seen and quantified. With the latter method, a huge number of sporozoites, ≈10^7^ to 10^8^, were observed (Figure, panels C, D). The inner structures were well preserved, nuclei were clearly visible, and we could observe apparent dividing forms (Figure, panel E). From the ticks collected from roe deer, only 2 tick salivary glands contained parasites; PCR products using Bab primers showed 100% identity with *Babesia* sp. EU1 (AY046575).

## Conclusions

Our study shows that *I. ricinus* ticks are competent vectors for *Babesia* sp*.* EU1. Not only can these ticks carry *Babesia* sp. EU1 DNA, but more importantly, they enable these parasites to complete their life cycle up to the production of infectious sporozoites. Direct invasion of erythrocytes by *Babesia* sp. EU1 undoubtedly classifies this species in the genus *Babesia*, a feature generally not proven for most *Babesia* spp.

The proportions of *Babesia* sp. EU1–infective ticks found in our study (3/223 from cattle farm and 2/31 from wild fauna reserve, not statistically different) are comparable to published prevalence of infected ticks (1%–2%) collected either from animals or vegetation (*6*–*8*,*14*,*15*). Whatever the biotope, *Babesia* sp. EU1 is always present, threatening also in anthropized zones (farming areas). Millions of parasites inside salivary glands were observed and could be injected to the vertebrate host, from the early stage of the tick feeding (11.7 mg) until repletion (277 mg), which represents a massive infection. These 2 epidemiologic features, combined with the increasing number of immunocompromised persons, should lead to more awareness of the risk related to this zoonotic pathogen.

*B.* *divergens* sporozoites were never seen in the salivary glands of adult *I. ricinus* ticks, even when ticks were collected from cattle. This finding is despite the large number of ticks examined (223), the prevalence of nymphs carrying *B.* *divergens* DNA collected from the farm pastures (87% in 2007 on 113 nymphs analyzed, data not shown), and the infectious status of the herd (serologic prevalence 56%). We therefore raise questions about the main transmitting stage (larvae, nymph, or adult?) and about the quantitative transmission of *B.* *divergens* by *I. ricinus* ticks (low number of produced and infectious sporozoites?). In Europe, human babesiosis could be caused by these 2 *Babesia* spp., each of which is transmitted by *I. ricinus* ticks but probably with different sporozoite-production features.
